# Circulating endocan and preeclampsia: a meta-analysis

**DOI:** 10.1042/BSR20193219

**Published:** 2020-01-07

**Authors:** Xia Lan, Zhaoming Liu

**Affiliations:** Department of Obstetrics, Chong Qing Health Center for Women and Children, Chongqing 401147, China

**Keywords:** endocan, meta-analysis, preeclampsia

## Abstract

**Background:** Endocan, a novel protein involved in inflammation and endothelial dysfunction, has been suggested to be related to preeclampsia, although the results of previous studies were not consistent. The aim of the study was to evaluate the potential difference of circulating endocan in women with preeclampsia and those with normal pregnancy.

**Methods:** Matched case–control studies evaluating the difference of circulating endocan between women with preeclampsia and those with normal pregnancy were identified via systematic search of PubMed and Embase databases. A random-effect model or a fixed-effect model was used to pool the results according to the heterogeneity. Subgroup analysis was performed to evaluate whether the timing of preeclampsia onset affected the outcome.

**Results:** Overall, eight matched case–control studies, including 451 women with preeclampsia and 442 women with normal pregnancy were included. Significant heterogeneity was detected among the included studies (*P* for Cochrane’s *Q* test = 0.006, *I*^2^ = 65%). Meta-analysis with a random-effect model showed that women with preeclampsia had significantly higher circulating level of endocan compared with women with normal pregnancy (standardized mean difference = 0.37, 95% confidence interval: 0.13–0.62, *P* = 0.003). Subsequent subgroup analyses showed that the difference of circulating endocan between women with early onset preeclampsia and those with normal pregnancy was not statistically different from that between women with late-onset preeclampsia and those with normal pregnancy (*P* for subgroup difference = 0.81).

**Conclusions:** Women with preeclampsia have higher circulating endocan than those with normal pregnancy.

## Introduction

Preeclampsia refers to a severe complication during pregnancy that is characterized of hypertension and proteinuria after 20 weeks of gestation in pregnant women with no evidence of previous hypertension [1–3]. Women with preeclampsia are at higher risk for the development of cardiovascular diseases during pregnancy and after delivery [4–6]. Moreover, preeclampsia has been identified as a risk factor of maternal and perinatal morbidity and mortality worldwide [1]. However, effective treatments for preeclampsia remain limited, and the only cure for preeclampsia may be premature delivery when it occurs [3]. In addition, since some prophylactic measures such as aspirin [7] have been proved to be effective to reduce the incidence preeclampsia if it is administered early (e.g. within 16 weeks of gestation), it is important for the early recognition of women who are at higher risk to develop preeclampsia during pregnancy [8,9].

The pathogenesis of preeclampsia is multifactorial [3]. According to previous studies, endothelial dysfunction and systematic inflammatory response are among the main pathophysiological mechanisms for preeclampsia [10–12]. Endocan, also known as endothelial cell-specific molecule 1 (ESM-1), is a circulation-detectable soluble dermatan sulfate proteoglycan that is secreted by vascular endothelial cells of many tissue, including placenta [13]. Accumulating studies suggest that endocan has been involved in angiogenesis, endothelial dysfunction, and inflammation, and accordingly, changes of circulating endocan has been observed in patients with cardiovascular diseases, including hypertension [14–16]. A recent study in a rat model of connective tissue disease related pulmonary arterial hypertension (CTD-PAH) showed that knockdown of endocan attenuated the severity of PAH and related cardiac dysfunction, accompanied with the inhibition of tumor necrosis factor-α (TNF-α) signaling pathways [17]. These results suggest that endocan may be a functional protein rather than a simple biomarker. Interestingly, it has also been suggested that change of circulating endocan may be a marker of women with preeclampsia [18–25]. However, results of previous pilot studies are inconsistent [18–25]. Some studies showed that circulating endocan may be higher in women with preeclampsia than those with normal pregnancy [18,20,22,24], while others did not find a significant difference regarding the circulating endocan between pregnant women with and without preeclampsia [19,21,23,25]. Since the sample sizes of these studies are generally small, some studies may be statistically underpowered to detect a significant difference of circulating endocan between women with preeclampsia and those with normal pregnancy. Therefore, in the present study, we performed a meta-analysis to synthesize the results of the previous studies in order to systematically evaluate the potential difference of circulating endocan in women with and without preeclampsia. Moreover, since it has been demonstrated that women with early- and late-onset preeclampsia may have different risk factors and outcomes [26], we also performed subgroup analyses to evaluate whether circulating endocan was changed in both the early- and late-onset preeclampsia compared with that in women with normal pregnancy.

## Methods

### Literature search

We followed the instructions of Meta-analysis Of Observational Studies in Epidemiology (MOOSE) guidelines [27] and the Cochrane’s Handbook for Systematic Review [28] throughout the design, implementation, analysis, and reporting for the present study. The electronic databases of PubMed and Embase were searched for relevant records, using the terms of ‘endocan’ OR ‘endothelial cell-specific molecule 1’ OR ‘ESM-1’, combined with ‘preeclampsia’ OR ‘pre-eclampsia’ OR ‘eclampsia’ OR ‘pregnancy-induced hypertension’ OR ‘PIH’ OR ‘toxemia’ OR ‘edema-proteinuria-hypertension gestos’ OR ‘EPH’. The search was limited to human studies published in English or Chinese. We also analyzed the reference lists of original and review articles using a manual approach. The final literature search was performed on June 25, 2019.

### Study selection

Studies were included for analysis if they met the following criteria: (1) published as full-length article; (2) included cases of women with preeclampsia and controls of women with normal pregnancy, at least matched for gestational age (GA); (3) serum or plasma level of endocan was measured in women with preeclampsia and women with normal pregnancy at the diagnosis of preeclampsia; and (4) reported circulating levels of endocan in cases and controls as means and standard deviations (SDs) or these data could be estimated. The diagnosis of preeclampsia was consistent with the American College of Obstetricians and Gynecologists criteria [29], which was classified as new onset (after 20 weeks of gestation) elevated systolic blood pressure ≥ 140 mm Hg and/or diastolic blood pressure ≥ 90 mm Hg and proteinuria (≥ 300 mg of protein in a 24-h urine collection); or as hypertension plus one of the following: (1) thrombocytopenia (platelet count <100,000 per microliter), (2) impaired liver function (twice the normal concentration of liver transaminases), (3) new renal insufficiency (>1.1 mg/dl or doubling of serum creatinine), (4) pulmonary edema and (5) new-onset cerebral or visual disturbances. Reviews, editorials, abstracts, preclinical studies or repeated reports of the same studies were excluded.

### Data extraction and quality assessment

Two authors performed the literature search, data extraction and quality assessment independently according to the inclusion criteria. Discrepancies were resolved by consensus. For each included study, data on first author’s name, year of publication, country of the study, maternal age, numbers of cases and controls, GA of blood sampling, and methods for endocan measurements were extracted. The study quality evaluation was performed according to the Newcastle–Ottawa Scale (NOS) [30], which varies from 1 to 9 stars, and 9 stars indicate the high quality of the study. This scale judges each study on three broad categories, including selection of the study groups; the comparability of the groups; and the ascertainment of the outcome of interest; 9 stars.

### Statistical analyses

Because different methods were used for the measurement of endocan in the included studies, and the values of endocan varied significantly (more than 10 folds) among these studies, standardized mean differences (SMD) with 95% confidence intervals (CI) were chosen as the measurement of difference for the circulating endocan in women with preeclampsia and in women with normal pregnancy as indicated by the Cochrane’s Handbook [28]. Inter-study heterogeneity was formally tested using Cochrane’s test, and significant heterogeneity was considered existing if *P* value was <0.10 [31]. The *I*^2^ statistic was also calculated, and a value of *I*^2^ > 50% indicated significant heterogeneity [32]. A random-effect model was applied to combine the data if significant heterogeneity was detected; otherwise, a fixed-effect model was used. Sensitivity analysis by omitting one study at a time was performed to evaluate the stability of the outcome [28]. Stratified analyses was performed to evaluate whether women with early- and late-onset preeclampsia both had higher circulating endocan compared with women with normal pregnancy. Predefined subgroup analyses were also performed to evaluate the influences of other study characteristics on the outcome, including study location, maternal age, sample size, type of blood sample, and NOS. Early-onset preeclampsia was defined as preeclampsia occurred at ≤33 weeks of gestation, while those occurred after 33 weeks of gestation was defined as late-onset preeclampsia [33]. The medians of continuous variables were chosen as the cutoff values for stratification. Potential publication bias was estimated by the visual inspection for the symmetry of the funnel plots complemented with the Egger’s regression test [34]. The statistical analysis was performed with RevMan software (Version 5.3; Cochrane Collaboration, Oxford, U.K.) and Stata software (version 12.0; Stata Corporation, College Station, TX, U.S.A.).

## Results

### Literature search results

**Figure 1:**
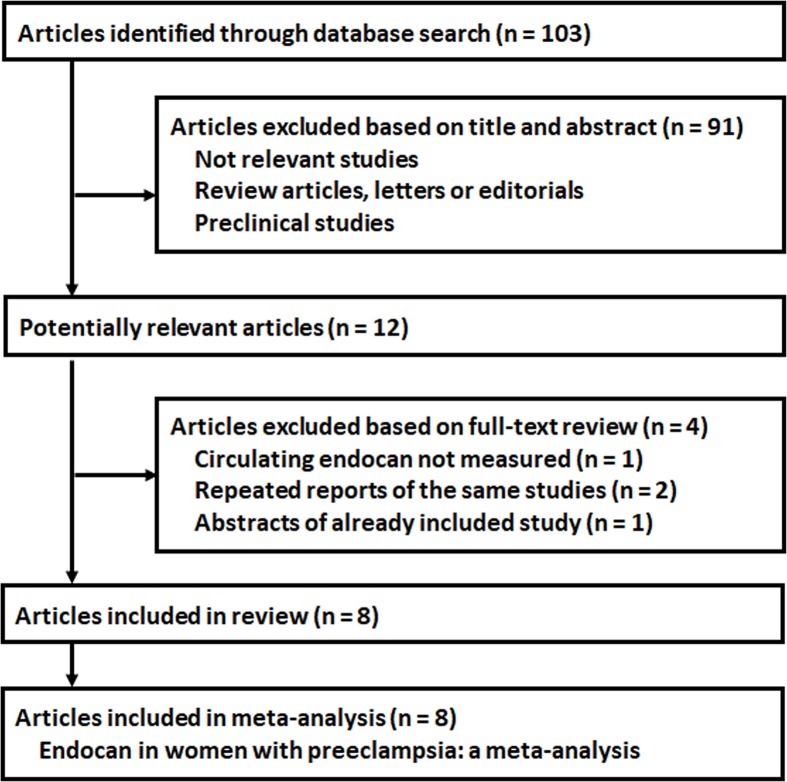
Flowchart of database search

The study selection process was shown in Figure 1. Overall, 103 citations were identified through initial database searching, of which 91 were excluded mainly because they were not relevant to the purpose of the study. The remaining 12 studies underwent full-text review, and eight studies were finally included [18–25]. The other four studies were excluded because circulating endocan was not measured in one study, two studies were repeated reports of the included studies, and another one was a conference abstract of an included study.

### Study characteristics

Three inconsistencies regarding data extraction and one inconsistency in quality evaluation occurred, and the consensus was achieved via reviewing the literature by the two authors together. The characteristics of included studies are shown in Table 1. All of the included studies were matched case–control studies published after 2015. The sample size of the included studies varied from 22 to 232. The mean maternal ages of the women varied from 24 to 31 years. In five studies, serum endocan was measured [19,21–23,25]; while for the remaining three studies, plasma endocan was measured [18,20,24]. For all the studies, an enzyme-linked immunosorbent assay (ELISA) was used to measure circulating endocan except for one study, of which a Milliplex Assay was applied [20]. The NOS of the included studies ranged between 7 and 8 stars.

**Table 1:** Characteristics of the included studies

Author	Country	Study design	Maternal age	Diagnosis of PE	No. of PE	No. of Control	GA at sampling	Endocan sample	Methods for endocan measurement	NOS
			Years				Weeks			
Yuksel 2015	Turkey	Matched CC	30.5	ACOG	49	32	At diagnosis (mean: 32)	Serum	ELISA	8
Hentschke 2015	Brazil	Matched CC	26	ACOG	50	67	At diagnosis (28–36)	Plasma	Milliplex assay	8
Chang 2015	China	Matched CC	29.9	ACOG	12	10	At diagnosis	Serum	ELISA	7
Adekola 2015	US	Matched CC	24.3	ACOG	102	130	At diagnosis	Plasma	ELISA	7
Cakmak 2016	Turkey	Matched CC	28.3	ACOG	99	30	At diagnosis (mean: 34)	Serum	ELISA	8
Wang 2017	China	Matched CC	29.7	ACOG	41	43	At diagnosis	Serum	ELISA	7
Schuitemaker 2018	the Netherlands	Matched CC	28.1	ACOG	38	51	At diagnosis (30–36)	Plasma	ELISA	8
Gozdziewicz 2019	Poland	Matched CC	31	ACOG	60	59	At diagnosis (30–38)	Serum	ELISA	8

Abbreviations: ACOG, American College of Obstetricians and Gynecologists; CC, case–control; ELISA, enzyme-linked immunosorbent assay; GA, gestational age; NOS, the Newcastle–Ottawa Scale; PE, preeclampsia.

### Circulating endocan in women with preeclampsia: main study and subgroup analyses

**Figure 2:**
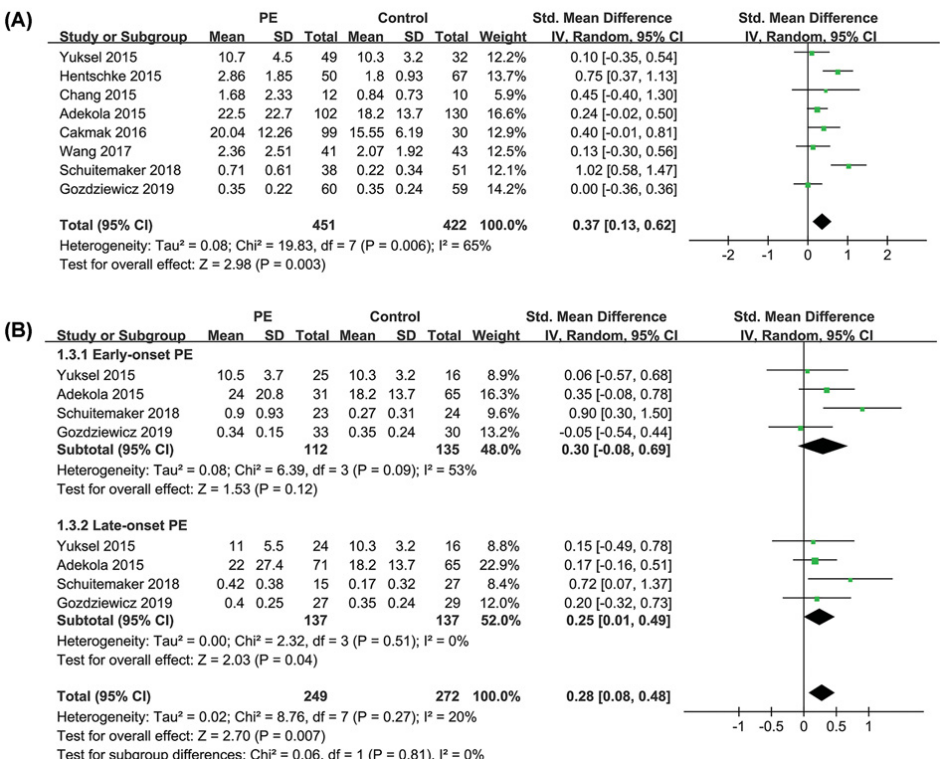
Forest plots for the meta-analysis Forest plots for the meta-analysis comparing circulating endocan between women with preeclampsia and those with normal pregnancy; (**A**) main analyses; and (**B**) stratified analyses by the timing of preeclampsia onset; PE, preeclampsia. For each figure, mean and SD indicate the mean values and SD of circulating endocan (μg/ml) in women from each group, and total indicate the total number of women in each group.

Overall, our meta-analysis included 451 women with preeclampsia and 442 women with normal pregnancy from eight case–control studies [18–25]. The heterogeneity among the included studies was significant (*P* for Cochrane’s *Q* test = 0.006, *I*^2^ = 65%). Results of meta-analysis with a random-effect model showed that women with preeclampsia had significantly higher circulating level of endocan compared with women with normal pregnancy (SMD = 0.37, 95% CI: 0.13–0.62, *P* = 0.003; Figure 2A). Sensitivity analyses by omitting data from one comparison at a time did not significantly change the results (SMD = 0.26–0.41, *P* < 0.05), indicating that the result of the meta-analysis was stable. Stratified analyses showed that circulating endocan was significantly higher in women with late-onset preeclampsia than those with normal pregnancy (*P* = 0.04) but the difference became insignificant when comparing between women with early-onset preeclampsia and normal controls (*P* = 0.12; Figure 2B). However, the difference between the subgroup was not significant (*P* = 0.81). Subsequent subgroup analyses showed that study characteristics such as study locations, sample sizes, types of blood samples, or NOS did not significantly affect the results (*P* for subgroup analyses all > 0.05; Table 2), while maternal age may significantly affect the outcome (*P* for subgroup analysis = 0.02). It seemed that the difference of circulating endocan between women with preeclampsia and normal pregnancy was mainly driven by studies with maternal age ≤ 29 years, but not for those with maternal age > 29 years (Table 2).

**Table 2:** Subgroup analyses for the difference between endocan between PE and normal pregnant women

Characteristics	Dataset number	SMD (95% CI) of pentraxin-3	*P* for subgroup effect	*I*^2^	*P* for subgroup difference
Study countries					
Others	6	0.41 [0.11, 0.70]	0.007	73%	
China	2	0.19 [-0.19, 0.58]	0.32	0%	0.39
Mean maternal age (years)					
≤29	4	0.58 [0.22, 0.93]	0.001	73%	
>29	4	0.09 [-0.13, 0.32]	0.42	0%	0.02
Sample size					
>100	4	0.42 [-0.06, 0.90]	0.09	72%	
≤100	4	0.34 [0.04, 0.63]	0.03	65%	0.77
Blood sample					
Plasma	3	0.65 [0.16, 1.13]	0.009	82%	
Serum	5	0.16 [-0.03, 0.36]	0.11	0%	0.07
NOS					
7	3	0.22 [0.01, 0.44]	0.04	0%	
8	5	0.45 [0.07, 0.83]	0.02	77%	0.30

Abbreviations: CI, confidence interval; NOS, the Newcastle–Ottawa Scale; PE, preeclampsia; SMD, standard mean difference.

### Publication bias

**Figure 3:**
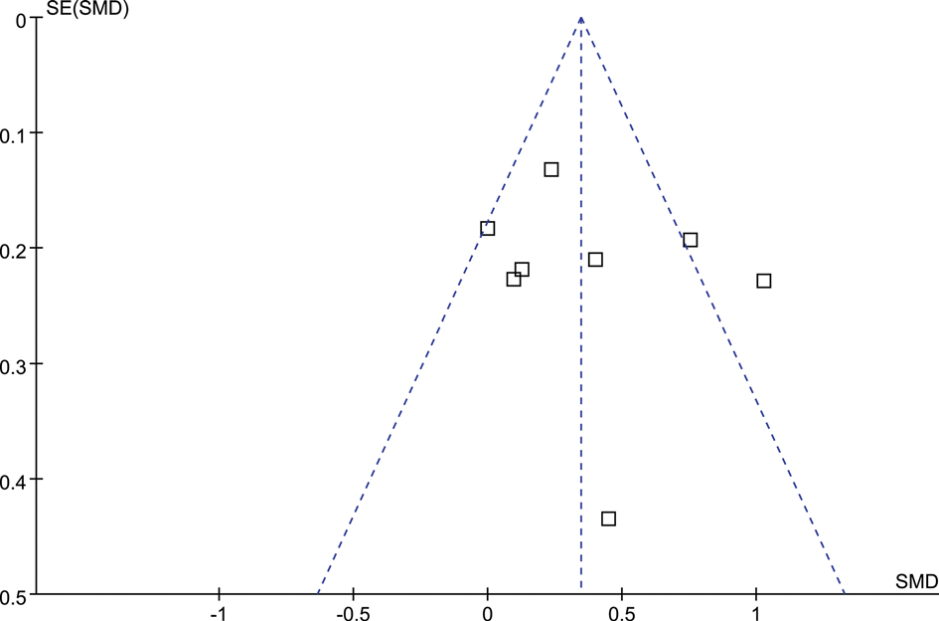
Funnel plots for the meta-analysis Funnel plots for the meta-analysis comparing circulating endocan between women with preeclampsia and those with normal pregnancy. Each box in the figure indicates an included study of the meta-analysis, and the plots were constructed by the effect size for each study (SMD) and its standard error (SE). The plots were symmetrical on visual inspection, suggesting low risk of publication bias for this meta-analysis.

The funnel plot was symmetrical on visual inspection, indicating low risk of publication bias (Figure 3). The result of Egger’s regression test also indicated no significant publication bias (*P* = 0.419).

## Discussion

In this meta-analysis, we found that women with preeclampsia have higher circulating endocan compared with women who are matched for gestational age with normal pregnancy. Subsequent analyses showed that study characteristics including the timing of study locations, sample sizes, types of blood samples, or study quality score did not significantly affect the outcome, while mean maternal age may have significant influence on the outcome. Stratified analyses showed that circulating endocan was significantly higher in women with late-onset preeclampsia than those with normal pregnancy, but the difference became insignificant when comparing between women with early-onset preeclampsia and normal controls. Moreover, results showed that the difference of circulating endocan between women with preeclampsia and normal pregnancy was mainly driven by studies including younger women (≤29 years), but not for those including older women (>29 years). Taken together, these results demonstrated that circulating endocan is higher in women with preeclampsia as compared with women with normal pregnancy. Future studies are needed to determine whether endocan was involved in the pathogenesis and progression or preeclampsia.

To the best of our knowledge, our meta-analysis is the first study to evaluate the potential difference of circulating endocan in women with preeclampsia and in women with normal pregnancy. We included matched case–control studies and the pooled results demonstrated that women with preeclampsia have higher circulating endocan than those with normal pregnancy. The robustness of the finding was further confirmed by the results of sensitivity and subgroup analyses, which showed that the outcome was not significantly affected by any single included study or study characteristics including study locations, sample sizes, types of blood samples, or study quality score. However, it remains undetermined whether up-regulated circulating endocan plays a role in the pathogenesis of preeclampsia. The initial pathophysiological change in preeclampsia is the impaired trophoblast invasion of the maternal spiral arteries [35]. The above change causes placental hypoxia, which further enhanced the overproduction and release of placenta derived anti-angiogenic and inflammatory factors, thereby contributing to the systematic manifestation of preeclampsia [3]. Endocan is an indicator of endothelial function, which is produced and secreted human endothelial cells of many organs [36]. Previous studies showed that endocan may participate in many processes related with endothelium, including cell adhesion, angiogenesis, inflammation and endothelial dysfunction [36], which have all been confirmed to be involved in the pathogenesis of preeclampsia [37–39]. Moreover, it has been shown that the endocan protein in placenta tissue is significantly up-regulated in women with preeclampsia [15,19], which also highlights the potential role of endocan in the pathogenesis of preeclampsia. To the best of our knowledge, no experimental studies have been performed to investigate the function of endocan in the pathogenesis of preeclampsia. Since it has been shown that knockdown of endocan attenuate the PAH related changes in a rat model of CTD-PAH via inhibition of TNF-α related inflammation pathways [17], it could be hypothesized that endocan may participate in the pathogenesis of preeclampsia via enhancing inflammation related endothelial dysfunction. Future studies are needed to determine the molecular pathways through that endocan may mediate the pathogenesis of preeclampsia.

Previous studies indicated that women with early-onset and late-onset preeclampsia may have different profiles of risk factors as well as different clinical outcomes [26]. A previous study showed that angiogenic factors are detectable before and at the time of clinical diagnosis of early-onset preeclampsia, whereas alterations were observed only at the time of diagnosis in women with late-onset preeclampsia [40]. Our stratified analyses showed that circulating endocan was significantly higher in women with late-onset preeclampsia than those with normal pregnancy, but the difference became insignificant when comparing between women with early-onset preeclampsia and normal controls, suggesting that timing of preeclampsia onset may affect the difference of circulating endocan between women with preeclampsia and normal pregnancies. Moreover, our subgroup analyses indicated that the difference of circulating endocan between women with preeclampsia and normal pregnancy was mainly driven by studies including younger women. This should be interpreted with caution since the variations for the age group in the included studies are limited, and the number of the included studies for each subgroup is relatively small.

Our study has some limitations which should be considered when interpreting the results. First, moderate heterogeneity was detected among the included studies. Although we performed subgroup analyses to explore the potential source of heterogeneity, it seems that none of the following study characteristics could adequately explain the potential heterogeneities, including study locations, sample sizes, types of blood samples, or study quality score. Since circulating endocan may be affected by factors which have impact on endothelial function, some remaining confounding factors may contribute to the heterogeneity, such as concurrent medications or some nutritional supplements during pregnancy. Second, it remains unknown whether up-regulated endocan participates in the pathogenesis of preeclampsia or it is simply just a marker of impaired endothelial function and activated inflammatory response. Third, future studies are needed to determine whether up-regulation of circulating endocan occurs before the onset of preeclampsia. Finally, a standardized protocol for measuring of circulating endocan has not been validated (from plasma or serum, the specific method, and the cut-off values). Therefore, it may be too early to propose that endocan could be used as a marker of preeclampsia at current state. However, results of our study demonstrated an increased circulating endocan in women with preeclampsia compared with those with normal control. Future studies are needed to determine whether endocan was involved in the pathogenesis and progression or preeclampsia.

In conclusion, results of meta-analysis showed that women with preeclampsia have higher circulating endocan than those with normal pregnancy. Future studies are needed to determine whether endocan was involved in the pathogenesis and progression or preeclampsia.

## Abbreviations

ACOG: American College of Obstetricians and Gynecologists
CC: case–control
CTD-PAH: connective tissue disease related pulmonary arterial hypertension
ESM-1: endothelial cell-specific molecule 1
GA: gestational age
NOS: Newcastle–Ottawa Scale
PE: preeclampsia
TNF-α: tumor necrosis factor-α
